# Curcumin and Colorectal Cancer: From Basic to Clinical Evidences

**DOI:** 10.3390/ijms21072364

**Published:** 2020-03-29

**Authors:** Maria Pricci, Bruna Girardi, Floriana Giorgio, Giuseppe Losurdo, Enzo Ierardi, Alfredo Di Leo

**Affiliations:** 1 THD S. p.A., 42015 Correggio (RE), Italy; mirellapricci@libero.it (M.P.); brunagirardi@virgilio.it (B.G.); flomic@libero.it (F.G.); 2 Gastroenterology Section, Department of Emergency and Organ Transplantation, University of Bari, 70124 Bari, Italy; giuseppelos@alice.it (G.L.); ierardi.enzo@gmail.com (E.I.)

**Keywords:** curcumin, colorectal cancer, cellular culture, animal model, cell proliferation, apoptosis

## Abstract

Curcumin diffuses through cell membranes into the endoplasmic reticulum, mitochondria, and nucleus, where it exerts actions, as an antioxidant property. Therefore, its use has been advocated for chemopreventive, antimetastatic, and anti-angiogenic purposes. We conducted a literature review to summarize studies investigating the relationship between curcumin and colorectal cancer (CRC). In vitro studies, performed on human colon cancer cell lines, showed that curcumin inhibited cellular growth through cycle arrest at the G2/M and G1 phases, as well as stimulated apoptosis by interacting with multiple molecular targets. In vivo studies have been performed in inflammatory and genetic CRC animal models with a chemopreventive effect. To improve curcumin bioavailability, it has been associated with small particles that increase its absorption when orally administered with excellent results on both inflammation and carcinogenesis. Curcumin has been used, moreover, as a component of dietetic formulations for CRC chemoprevention. These combinations showed in vitro and in vivo anticarcinogenetic properties in inflammation-related and genetic CRC. A synergic effect was suggested using an individual constituent dosage, which was lower than that experimentally used “in vivo” for single components. In conclusion, curcumin falls within the category of plant origin substances able to prevent CRC in animals. This property offers promising expectations in humans.

## 1. Introduction

Curcumin is a phytochemical derived from turmeric (Curcuma longa) which is a plant similar to ginger [1]. From a chemical point of view, it is a natural phenol with a typical yellow color. It is easily soluble in acetic acid, ketone, alkali, and chloroform, while it is insoluble in water at acidic and neutral pH [2]. Due to its hydrophobic properties, it is able to diffuse through cell membranes into the endoplasmic reticulum, mitochondria, and nucleus; in all these sites it can exert its action [3]. Curcumin is commonly utilized as an element of dietary supplements, a component of cosmetics, and a flavoring for foods and beverages especially in South and Southeast Asia. However, curcumin has a known antioxidant property, therefore its use has been advocated for chemopreventive, antimetastatic, and anti-angiogenic purposes [4].

Colorectal cancer (CRC) is one of the most widespread tumors worldwide, and it is considered to be the second leading cause of death among cancer groups [5]. The peak in the incidence of CRC in Western countries could be related to changes in lifestyle, and in particular, shifts in dietary habits could explain such a trend [6,7]. Indeed, the development of colonic carcinogenesis is highly influenced by environmental factors, notably carcinogenesis of the alimentary tract. From this perspective, a diet rich in polyunsaturated fats and red meat and poor in vegetables has been considered to be a major risk factor for CRC [8]. For this reason, modulation of diet composition could be invoked as a strategy to chemically prevent the onset of CRC, and curcumin could be an attractive food constituent. Indeed, it has been suggested as an anticarcinogenetic agent for several tumors, including prostate, pancreas, breast, stomach, liver carcinomas, and leukemia [9,10,11]. Among the proposed mechanisms of action, induction of epithelial apoptosis seems to be the most investigated [12]. Indeed, it has been shown that curcumin can promote the synthesis of proteins related to apoptotic processes and could interplay with the pathways of inflammation-related programmed death [13,14,15,16].

On the basis of this, the use of curcumin in chemoprevention and as a complementary treatment of CRC is promising. Herein, we carried out a literature review that aims to summarize the studies investigating the relationship between curcumin and CRC in vitro, animal models, and human trials.

## 2. In Vitro Studies: The Effect of Curcumin on Colon Cancer Cell Lines

Curcumin has been reported as an agent that is able to prevent CRC growth by blocking the cell cycle and accelerating apoptosis. In vitro studies, performed on different human colon cancer cell lines, showed that curcumin significantly inhibited cell growth by interacting with multiple molecular targets, thus, resulting in the modulation of several distinct signaling pathways.

In the human colon cancer cell line HCT-116, Mosieniak et al. demonstrated that curcumin inhibited cell proliferation by cell cycle arrest at the G2/M phase, and partially the G1 phase [17]. Moreover, Lim et al. [18] found that curcumin negatively regulated cyclin D1 and induced cell cycle interruption at the G1 phase in the same colon cancer cell line. Cyclin D1 is known to bind both CDK4 and CDK6, thus, forming an active complex which further phosphorylates Rb protein at Ser780 and regulates the transition from G1 to S phase as a final result [19].

A study performed by Kim and Lee revealed that curcumin inhibited cell proliferation of HCT-116 through the induction of reactive oxygen species (ROS) generation, and downregulation of E2F4 and related genes, such as cyclin A, p21, and p27 [20]. Another study by Watson et al. about curcumin cytotoxicity on HCT-116 and HT29 cell lines revealed that a sequential time and dose dependent inhibition of cell proliferation was observed when p53 was upregulated [21].

As reported above, one of the main mechanisms through which curcumin blocks cell growth is the induction of apoptosis. This process in the CRC cells involves multiple molecular targets including enzymes (cyclooxygenase-2 (COX 2)), transcription factors (NF-kB and beta-catenin), Bcl-2 family members (Bcl-2, Bax, and Bcl-xL), death receptors (death receptor 5 (DR5) and Fas), protease enzymes (caspase 3 and caspase 8), and ROS.

A COX-2 increased expression has been seen in many tumors, including CRC [22]. In detail, an enhanced expression of COX-2 was detected in 77% of cases of CRC as compared with the normal surrounding mucosa [23]. Further evidences demonstrated that curcumin downregulated COX-2 expression in CRC [24,25]. Moreover, curcumin exerted apoptotic effects on HT-29 colon cancer cell lines by means of COX-2 and apoptosis-related pAKT kinase reduction, as well as by increased p-AMP protein kinase (AMPK) signal [26].

NF-kB has been extensively investigated due to its involvement in CRC [27]. In this regard, curcumin can reduce the expression of NF-kB in CRC cells [28]. Collect and Campbell [29] described that curcumin promoted apoptosis in the HCT-116 line through the inhibition of NF-kB.

Beta-catenin transcription factor plays a critical role in the pathogenesis of CRC due mainly to APC inactivation and beta-catenin mutations. Both processes promote beta-catenin nuclear accumulation and transcription of many oncogenes [30]. Beta-catenin is the key nuclear effector of the well-recognized Wnt signaling in the nucleus and the integral structural component of cadherin-based adherens junctions [31]. A study performed by Narayan on human colon cancer cells showed that curcumin inhibited the Wnt/beta-catenin pathway by suppressing c-myc expression and inducing caspase 3 mediated cleavage of beta-catenin, E-cadherin, and APC. All these processes are linked to apoptosis and the G2/M phase arrest in HCT-116 colon cancer cells [32]. Finally, a study by Park et al. on both colon cancer SW480 and HCT-116 cell lines reported that curcumin inhibited the beta-catenin/Tcf signaling through decreased levels of nuclear beta-catenin and Tc-4 protein [33].

Many cancer histotypes, including CRC, have been associated with an impaired expression of Bcl-2 family molecules [34]. It has been shown that curcumin promotes Bax expression and reduces Bcl-2 in colon adenocarcinoma all through the phosphorylation at Ser15 and activation of p53 [35] Enhanced Bax expression could influence Bcl-2/Bax or Bcl-xL ratio, thus driving neoplastic cells to apoptosis. Curcumin-induced inhibition of Bcl-2 and upregulation of Bax have been reported even in other colon cancer lines like HCT-116 [36] and COLO-205 [37].

Death receptors, such as DR5 or Fas, play a pivotal role in the transmission of death signal from the cellular membrane to the cytoplasmic signaling pathways [38]. It has been reported that curcumin can upregulate DR5 protein, which is a receptor which is fundamental for apoptosis in HCT-116 and HT-29 colon cancer cells [39]. Furthermore, curcumin was discovered to trigger caspase 8 activation, which is a process that initiates Fas-mediated apoptotic pathway [40]. Procaspase 8 generates with Fas ligand a complex that constitutes the death-inducing signaling complex (DISC), thus activating caspase 8 by mutual splitting and promoting caspase 3, caspase 7, and Bid.

Curcumin plays its cytotoxic effect by also producing reactive oxygen species (ROS). Although curcumin is a powerful scavenger of free radicals, there are evidences that have also showed its possible role in promoting the generation of free radicals [41]. Curcumin has been found to trigger apoptosis by enhancing ROS production, hence inducing oxidative reactions and lysis of mitochondrial membranes in CRC cancer cells [42].

In conclusion, the anticancer effect of curcumin can be mediated by several mechanisms, which result in reduced cell growth and increased apoptosis. All these studies strongly encourage attempting to translate in vivo to what was observed in vitro.

## 3. In Vivo Studies: The Effect of Curcumin on Colorectal Cancer in Animal Models

In an animal model (mouse), Perkins et al., in 2002 [43], demonstrated that an intake of curcumin at 0.2%, which corresponded to 300 mg/kg, prevented or delayed adenoma development. In detail, small- and medium-sized adenomas were most sensitive to the chemopreventive effect of curcumin. The reduction of the number of adenomas was more evident in the central and distal regions of the intestinal tract. This study used C57BL/6J Min/+ (ApcMin/+) mice for a model simulating human familial adenomatous polyposis (FAP). The mice received a standard diet enriched with curcumin at concentrations of 0.1%, 0.2%, and 0.5% for 15 weeks. The substance at 0.1% did not show any effect, whereas concentrations of 0.2% and 0.5%, reduced the intestinal tumor number significantly by 39% and 40%, respectively. Successively, Park et al. positively commented on the relevance of this result [44].

Moreover, McFaden et al. [45] used a well-documented model of a study on the development of colitis-associated CRC. This study was performed using specific pathogen-free wild-type (WT) 129/SvEv mice and germ-free interleukin (IL)10−/− mice. Starting from 10 weeks of age, WT or IL10−/− mice received once weekly intraperitoneal injections of azoxymethane (AOM) or saline for six weeks, and, simultaneously, started a curcumin-supplemented diet. This study showed several highlights. This study demonstrated an almost complete reduction of CRC burden in IL-10 KO/AOM mice. These chemopreventive effects appeared to be indirectly related to a normalizing effect of curcumin on colonic microbial flora more than its anti-inflammatory effect. Interestingly, the study suggested the property of restoring the healthy gut homeostasis and microbial-host relationship. However, all transgenic mice experiencing AOM and treated with curcumin died, whilst only a 50% mortality was seen in the control group. Furthermore, the mice fed with curcumin tended to eat less and lose weight. Nevertheless, curcumin entirely prevented body weight loss in AOM-treated IL10−/− mice without a difference from the AOM-treated WT mice on the control diet. This event could have impacted on their AOM uptake and effect, as well as microbiota composition. Additionally, no polypoid lesions were observed in the AOM-treated WT mice exposed to curcumin. Finally, it normalized the beta-catenin expression pattern in the colonocytes. Of note, the two different housing types (IL10-KO and wild-type) made the comparison of results quite difficult, although they were treated with the same protocol.

Epigenetic mutations are of great interest for their role in carcinogenesis. DNA methylation is a common epigenetic mechanism associated with aberrant gene expression in cancer. Epigenetic modifications are highly associated with dietary factors [46]. In this regard, Yue Guo et al. [47] studied DNA methylome and transcriptome alterations and cancer prevention by curcumin in colitis-associated CRC in mice. Four-week-old male C57BL/6 wild-type mice received a chemical treatment of AOM and dextran sulphate sodium (DSS). They were fed a specific diet containing curcumin from 5 weeks of age until the end of the experiment. Decreased methylation was observed. Additionally, the curcumin attenuated colon shortening due to fibrosis consequent to long standing inflammation. Adenoma or adenocarcinoma were not observed in the group that consumed the AOM/DSS curcumin as compared with the animals fed a standard diet.

As reported above, curcumin is poorly water soluble and its bioavailability may be low due to the extreme conditions of the intestinal tract. To overcome these drawbacks, it has been recently associated with substances or small particles that allow transporting and increasing its absorption. [48]. For this purpose, Han et al. [49] tried out orally deliverable nanotherapeutics by combining water-insoluble curcumin and 7-ethyl-10-hydroxycamptothecin (SN38) which is the active metabolite of irinotecan. They studied the effects of this formulation on inflammatory bowel disease (IBD) and CRC in a mouse model. Compared with the individual drug form, the combination of curcumin and SN38 nanoparticles exerted synergistic beneficial effects on intestinal inflammation. The effects could be due to the synergistic effect of SN38-curcumin, rather than to the improved drug absorption. Of interest, oral administration of the combined formulation by drinking water was more effective than parenteral injection. The disease activity index, body weight loss, stool consistency, and intestinal bleeding, as well as mortality were reduced by curcumin-SN38. Simultaneously, the tumor number per mouse, as well as diameter and size were much reduced and histological analysis revealed that the majority of polypoid lesions were adenomas with low-grade dysplasia. The combination of curcumin and SN38 inhibited the progression of CRC acting on cell cycle and apoptosis. Indeed, cyclin D1 and D3 were significantly downregulated and proapoptotic proteins (cleaved forms of caspase 3, 7, 9 and cleaved-PARP) upregulated by curcumin and SN38. Finally, a reduction of anti-apoptotic Bcl-2 protein expression was seen.

**Figure 1:**
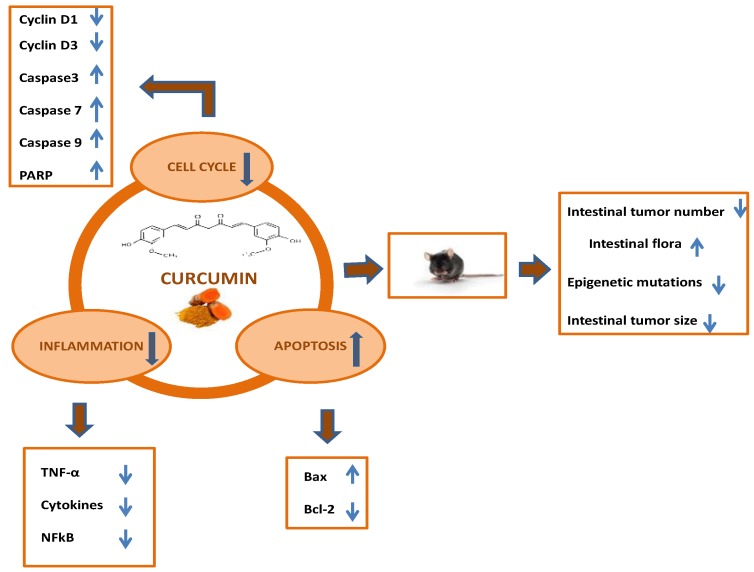
Chemoprevention of colorectal cancer by curcumin in animal models: Main mechanistic pathways.

The main mechanisms by which curcumin can prevent colorectal cancer development in animal models are summarized in Figure 1.

## 4. Effect of Curcumin as a Component of Dietetic Formulations of Plant Origin

Several molecules derived from plants have been described to have an effect in reducing intestinal cancer onset, in particular in animal models. Silymarin can hamper intestinal carcinogenesis by both antioxidant and estrogen receptor-beta (ER-beta) agonist functions [50,51]. Additionally, boswellic acids and especially acetyl-11-keto-beta-boswellic acid (AKBA) are constituents of gum resin of *Boswellia serrata* and they are considered to be promising agents for gut carcinogenesis prevention [52].

On the basis of the possibility that a phytochemical combination could exert beneficial effects under what was provided by a single plant substance [53], the effect of every component of a nutritional combination of silymarin, ABKA, and curcumin was compared “in vitro” with the complete mixture on cultured cancer cell proliferation (DLD-1). Each substance showed a relevant antiproliferative effect on colonic cancer cultured cells as compared with a control sample. Moreover, the effect of the mixture of the three components was much higher than their single or double combination [54].

Then, this enriched nutritional formulation was tested to prevent inflammation-associated CRC in an AOM/DSS animal model [54]. Anti-inflammatory and chemopreventive effects were estimated by lesion number and size, as well as by the detection of histological inflammation, dysplastic, and neoplastic areas. In addition, proinflammatory cytokine mRNA molecular pattern, ER-beta and bromodeoxyuridine immunohistochemistry, and TUNEL immunofluorescence labeling have been performed. Enriched, but not standard formulation, prevented the shortening of the colon (a hallmark of a long-standing inflammation). Moreover, dietetic formulation reduced polypoid lesion number and size, histological inflammation score, proinflammatory cytokine mRNA expression, and the number of low- (LGD) and high-grade dysplasia areas. Finally, CRC was observed in 69.6% of the standard and 23.5% of the enriched formulation consuming animals. The enriched formulation induced higher ER-beta expression in LGD and increased apoptosis in LGD. For the well-known anticancer effect of ER-beta, this study suggested that LGD could represent the checkpoint for neoplastic evolution and ER-beta agonist function of supplemental formulation could promote apoptosis, thus slowing the progression towards carcinoma. Indeed, the concurrent increased TUNEL expression, reported in LGD, suggested a direct relationship between ER-beta and apoptosis. This result is in agreement with what was previously found by our group, i.e., the colocalization of ER-beta and caspase 3, which is a recognized early apoptotic marker [55,56]. Furthermore, the epithelial cell migration was observed in the normal epithelium from the base of the crypt to the top of the villi, in order to evaluate the effect of dietetic formulation on this physiological process. The finding that epithelial migration in normal tissue was faster in the enriched as compared with the standard diet group suggested a reduced cellular half-life, which decreased the time of exposure of DNA synthesizing cells to mutation risk. Indeed, the reduced half-life of cells led to an accelerated turnover and, therefore, to an early cellular death, which are all events that prevent the tumor growth and the accumulation of DNA mutations that promote carcinogenesis.

**Figure 2:**
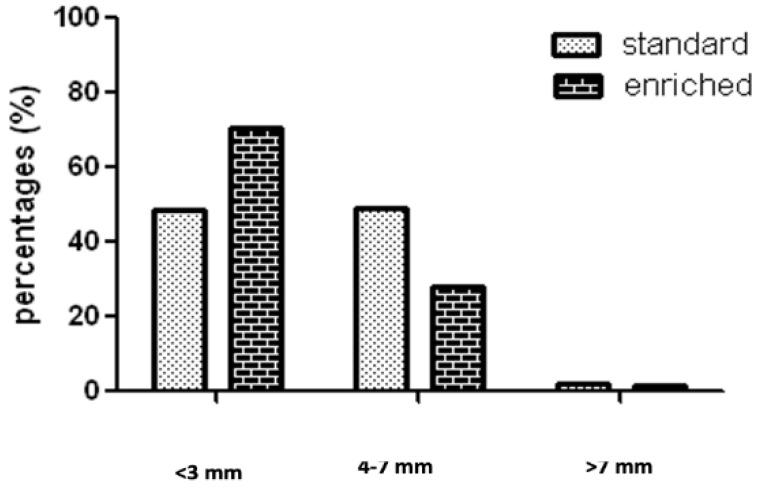
Percentage of small (<3 mm), intermediate (3–7 mm) and large (>7 mm) polypoid lesions in ApcMin/+ mice assuming standard or enriched diet. Statistical analysis (Chi square for trend, *p* < 0.001).

The chemopreventive effects of the same dietetic formulation was investigated also in ApcMin/+ mice [57]. Compared to standard diet, the enriched diet reduced the total and mean number of polypoid lesions as well as areas of LGD and CRC. In addition, the polyp size was significantly reduced in the enriched diet group (Figure 2). The ER beta protein showed a marked signal associated with dietetic supplementation, and in normal mucosa, cleaved caspase 3 showed a stronger signal in the enriched than in the standard diet. This result confirmed the strong relationship between ER beta and apoptosis. Cyclin D1 (marker of cell proliferation) was more expressed in the standard than the enriched diet group of both the normal and the polypoid tissue. Epithelial migration in normal tissue showed a pattern similar to the AOM/DSS model previously described. The effect of dietary formulation intake appeared to be mediated by the reduction of epithelial proliferation, the increase of apoptosis, and the acceleration of villous cell renewal with a reduced risk of DNA mutations.

These studies suggest the potential usefulness of the combination of different phytochemicals in CRC chemoprevention in animal models. The advantage of the mixture seems to be related to a synergic effect suggested by the dosage used for the individual nutritional components, which turns out to be lower than that used for the single substances for “in vivo” experiments on the same animal models [43,58,59].

## 5. Human Clinical Studies

Curcumin has been tested in early preclinical studies in order to evaluate the best tolerated dose. Indeed, it is known that curcumin at a high dose or with prolonged exposure can cause hepatobiliary adverse events by interfering with colecistokinin signaling.

Storka et al. [60] tested curcumin safety at doses ranging from 10 to 400 mg/m2. Liposomial curcumin at 120 mg/m^2^ was the best tolerated dose and avoided the appearance of, and increase in, the mean red blood cell volume in the blood, observed at higher dosages.

Other preclinical studies aimed to ascertain whether, after curcumin ingestion, some of its metabolites (curcuminoids) could be found in colonic epithelial tissues. For instance, echinocytes in a group of 26 subjects consuming 2.35 g/day for 14 days, curcuminoids were found in 28 out of 35 biopsy samples, thus, confirming that curcumin is absorbed and binds to colonocytes [61].

Curcumin has also been tested in healthy subjects to evaluate its protective effect against oxidative stress. In this regard, it has been demonstrated that a dose of 3.6 g/day was able to reduce DNA adducts on colon biopsy samples [62]. Similarly, it was given to smokers at dosages of either 2 g or 4 g/day for 30 days showing that only a high dose was able to reduce the number of aberrant crypt foci in the colon [63]. The same study, however, failed to find a reduction of proliferation epithelial index by Ki67.

Additionally, some applications of curcumin for patients with inherited polyposis, such as familial adenomatous polyposis (FAP) have been described. In a small group of five FAP patients with rectal remnant after subtotal colectomy, Cruz-Correa et al. [64] administered a mixture of curcumin 480 mg plus quercetin 20 mg t.i.d for six months, thus, demonstrating by endoscopy a 60% reduction in polyp number and a 50% reduction in polyp size. Successively, the same group, in a randomized placebo-controlled trial, tested a high dose (1500 mg b.i.d) for one year in 44 FAP patients [65]. Surprisingly, a difference in polyp size and number between placebo and curcumin was not found. Furthermore, a case report [66] of a patient with 54 polyps at index colonoscopy, with all genetic tests negative for genetic polyposis demonstrated an interesting result. Indeed, after the removal of about 40 polyps, the patient was given curcumin 400 mg/day for three months. After about two years, only three polyps were detected at surveillance colonoscopy.

Finally, curcumin has been used as an adjunctive treatment to traditional chemotherapy for advanced colorectal carcinoma with promising results. For instance, patients undergoing FOLFOX regimen were randomized to curcumin 2 g/day or no supplementation for 12 cycles [67]. This was a phase II open-labelled randomized controlled trial which showed an improved overall survival in the curcumin group, despite no improvement in quality of life or neurotoxicity was found. A similar study is ongoing [68]; in this case patients with inoperable CRC will be randomized to curcumin versus placebo in addition to FOLFOX regimen. In this case, various doses of curcumin between 0.5 and 2 g/day will be used. Finally, a phase I study on a small group of patients with metastatic cancer consuming liposomal curcumin (300 mg/m twice weekly for 8 weeks) did not show any antitumoral activity in reducing cancer size according to RECIST criteria [69]. In conclusion, the analyzed studies do not provide univocal results. However, we believe that most of the negative results have been observed in extreme conditions, i.e., patients with advanced cancer and patients in which the genetic burden is very high (FAP subjects). Therefore, it is likely that a phytochemical could not exert a sufficiently strong effect to overcome these conditions. As a consequence, studies performed on subjects with generic risk of CRC with the aim of proving the effect on precancerous lesions could supply more interesting conclusions. The results provided by such studies are reported in Table 1.

## 6. Side Effects of Curcumin

The safety of curcumin has been extensively investigated. Turmeric extracts and curcumin have not shown any major toxic effects when given to rodents; in addition, no mutagenic or genotoxic effects have been observed in pregnant animals [70]. Moreover, oral administration of curcumin for 70 days, at doses up to 10,000 ppm have been found not to be toxic in rats [71]. According to animal studies, standardized fine particles and extract of curcumin have been demonstrated to be safe for human use even at high doses (1.5 g/day curcumin) and for periods up to six months [70].

Five randomized controlled trials have described adverse events. Rahmani et al. [72] reported that two patients had simultaneously abdominal pain and nausea, and another patient complained only of abdominal pain. Amin et al. [73] reported some side effects such as nausea and dyspepsia, although the precise number of subjects who encountered these events was not detailed. In a report by Chuengsamarn et al. [74], the following curcumin-related adverse events were revealed in four subjects: constipation (two cases), hot flashes (one), and nausea (one). Nevertheless, in the last study, four subjects reported the following side effects in the placebo group: vertigo and itching, constipation, and hot flashes each in one patient. Selvi et al. [75] found mild diarrhea in two cases.

Twelve systematic reviews reported adverse effects, which were classified as mild and similar to a placebo. The most frequent adverse events included abdominal pain, nausea, and dyspepsia [76].

Of interest, Medina-Caliz et al., in an analysis of cases of herbal and dietary supplement-induced liver injury in Spain, found that herbal and dietary supplements were responsible for 4% (32 cases) of the 856 drug induced liver injuries [77]. Such events occurred more frequently among young women and were associated with hepatocellular injury and a rise of transaminase levels. Herbal and dietary supplements caused a more severe liver injury than that observed in other types of drug-induced hepatic damage. Additionally, the recurrence of liver malfunction was more likely after a second re-exposure. Incidentally, Iman et al. described a case of curcumin-induced hepatocellular damage in a 78 year old woman admitted with jaundice, with a latency time of one month [78]. Laboratory investigations failed to find any other cause of acute hepatitis. The Roussel Uclaf Causality Assessment Method (RUCAM) score was six, suggesting a probable association. Peak levels of aspartate aminotransferase (AST) and alanine aminotransferase (ALT) were more than 20 times the normal upper limit. A halving of AST and ALT was seen one week after supplement withdrawal, while reversal of transaminases peak was reported after 42 days. No rechallenge was performed.

In conclusion, curcumin appears to be safe, but long-term studies, especially in children and adolescents or pregnant women, as well as trials that focus on nanoformulations are necessary to completely support its security.

## 7. Conclusions

In conclusion, several evidences demonstrate that curcumin falls within the category of plant origin substances able to prevent CRC. Different possible mechanisms have been demonstrated in studies performed both in vitro and in vivo in animal models. Additionally, evidence of clinical benefits have been observed in mice with inflammatory and genetic CRC. Curcumin has been administered alone or associated with substances or small particles that allow transporting and increasing its absorption when orally administered. Furthermore, curcumin has been used as a component of dietetic formulations of substances of plant origin. This property offers promising expectations in humans. Nevertheless, there is no clear evidence that the results obtained on cultured cells or animal models can be translated in humans. Indeed, on the one hand, human clinical studies are very few and have shown controversial results. On the other hand, open questions regarding dosage, bioavailability, optimal indication, and potential toxicity need to be clarified in future studies with large samples.

## Figures and Tables

**Table 1:** The main human studies that investigating curcumin effect in colorectal cancer.

Study	Type of Study	Main Results
Storka et al., 2015 [60]	Safety study	120 mg/m^2^ was the best tolerated dose. Echinocytes appeared at higher doses
Irving et al., 2013 [61]	In vivo study to verify ability of curcumin to be absorbed in colonocytes	Curcuminoids were found in 28 out of 35 biopsy colon samples
Garcea et al., 2005 [62]	In vivo study to explore antioxidant properties	Reduction of DNA adducts
Carroll et al., 2011 [63]	Evaluation of dysplasia after curcumin administration	Reduction of the number of aberrant crypt foci in the colon
Cruz-Correa et al., 2006 [64]	FAP patients receiving curcumin	60% reduction in polyp number and a 50% in polyp size at endoscopy
Cruz-Correa et al., 2018 [65]	FAP patients receiving curcumin	No difference in polyp size and number between placebo and curcumin
Alfonso-Moreno et al., 2017 [66]	Case report of a patient with sporadic polyposis	Reduction in polyp number at surveillance endoscopy
Howells et al., 2019 [67]	Curcumin as adjuvant regimen to FOLFOX	Improved overall survival in the curcumin group
Greil et al., 2018 [69]	Phase I study on patients with metastatic cancer	No variation in tumor size according to RECIST criteria

## References

[1] Shehzad A., Wahid F., Lee Y.S. (2010). Curcumin in cancer chemoprevention: Molecular targets, pharmacokinetics, bioavailability, and clinical trials. Arch. Der. Pharm..

[2] Chattopadhyay I., Biswas K., Bandyopadhyay U., Banerjee R.K. (2003). Turmeric and Curcumin: Biological actions and medicinal applications. Curr. Sci..

[3] Jaruga E., Sokal A., Chrul S., Bartosz G. (1998). Apoptosis-independent alterations in membrane dynamics induced by curcumin. Exp. Cell Res..

[4] Aggarwal B.B., Sung B. (2009). Pharmacological basis for the role of curcumin in chronic diseases: An age-old spice with modern targets. Trends Pharmacol. Sci..

[5] Fitzmaurice C., Allen C., Barber R.M., Barregard L., Bhutta Z.A., Brenner H., Dicker D.J., Chimed-Orchir O., Dandona R., Dandona L. (2017). Global, Regional, and National Cancer Incidence, Mortality, Years of Life Lost, Years Lived with Disability, and Disability-Adjusted Life-years for 32 Cancer Groups, 1990 to 2015: A Systematic Analysis for the Global Burden of Disease Study. JAMA Oncol..

[6] Castelló A., Amiano P., Fernández de Larrea N., Martín V., Alonso M.H., Castaño-Vinyals G., Pérez-Gómez B., Olmedo-Requena R., Guevara M., Fernandez-Tardon G. (2019). Low adherence to the western and high adherence to the mediterranean dietary patterns could prevent colorectal cancer. Eur. J. Nutr..

[7] Barone M., Lofano K., De Tullio N., Licinio R., Albano F., Di Leo A. (2012). Dietary, endocrine, and metabolic factors in the development of colorectal cancer. J. Gastrointest. Cancer.

[8] Alexander D.D., Weed D.L., Miller P.E., Mohamed M.A. (2015). Red Meat and Colorectal Cancer: A Quantitative Update on the State of the Epidemiologic Science. J. Am. Coll. Nutr..

[9] Aggarwal B.B., Kumar A., Bharti A.C. (2003). Anticancer potential of curcumin: Preclinical and clinical studies. Anticancer Res..

[10] Lopez-Lazaro M. (2008). Anticancer and carcinogenic properties of curcumin: Considerations for its clinical development as a cancer chemopreventive and chemotherapeutic agent. Mol. Nutr. Food Res..

[11] Hanif R., Qiao L., Shi S.J., Rigas B. (1997). Curcumin, a natural plant phenolic food additive, inhibits cell proliferation and induces cell cycle changes in colon adenocarcinoma cell lines by a prostaglandin-independent pathway. J. Lab. Clin. Med..

[12] Ismail N.I., Othman I., Abas F., Lajis N., Naidu R. (2019). Mechanism of Apoptosis Induced by Curcumin in Colorectal Cancer. Int. J. Mol. Sci..

[13] Agarwal A., Kasinathan A., Ganesan R., Balasubramanian A., Bhaskaran J., Suresh S., Srinivasan R., Aravind K.B., Sivalingam N. (2018). Curcumin induces apoptosis and cell cycle arrest via the activation of reactive oxygen species-independent mitochondrial apoptotic pathway in Smad4 and p53 mutated colon adenocarcinoma HT29 cells. Nutr. Res..

[14] Su P., Yang Y., Wang G., Chen X., Ju Y. (2018). Curcumin attenuates resistance to irinotecan via induction of apoptosis of cancer stem cells in chemoresistant colon cancer cells. Int. J. Oncol..

[15] Bush J.A., Cheung K.J., Li G. (2001). Curcumin induces apoptosis in human melanoma cells through a Fas receptor/caspase 8 pathway independent of p53. Exp. Cell Res..

[16] Plummer S.M., Holloway K.A., Manson M.M., Munks R.J., Kaptein A., Farrow S., Howells L. (1999). Inhibition of cyclooxygenase 2 expression in colon cells by the chemopreventive agent curcumin involves inhibition of NF-kB activation via the NIk/Ikk signaling complex. Oncogene.

[17] Mosieniak G., Adamowicz M., Alster O., Jaskowiak H., Szczepankiewicz A.A., Wilczynski G.M., Ciechomska I.A., Sikora E. (2012). Curcumin induces permanent growth arrest of human colon cancer cells: Link between senescence and autophagy. Mech. Ageing Dev..

[18] Lim T.G., Lee S.Y., Huang Z., Lim D.Y., Chen H., Jung S.K., Bode A.M., Lee K.W., Dong Z. (2014). Curcumin suppresses proliferation of colon cancer cells by targeting CDK2. Cancer Prev. Res..

[19] Hernando E., Nahle Z., Juan G., Diaz-Rodriguez E., Alaminos M., Hemann M., Michel L., Mittal V., Gerald W., Benezra R. (2004). Rb inactivation promotes genomic instability by uncoupling cell cycle progression from mitotic control. Nature.

[20] Kim K.C., Lee C.H. (2010). Curcumin induces downregulation of E2F4 expression and apoptotic cell death in HCT116 human colon cancer cells; involvement of reactive oxygen species. Korean J. Physiol. Pharmacol..

[21] Watson J.L., Hill R., Yaffe P.B., Greenshields A., Walsh M., Lee P.W., Giacomantonio C.A., Hoskin D.W. (2010). Curcumin causes superoxide anion production and p53-independent apoptosis in human colon cancer cells. Cancer Lett..

[22] Wu Q.B., Sun G.P. (2015). Expression of COX-2 and HER-2 in colorectal cancer and their correlation. World J. Gastroenterol..

[23] Roelofs H.M., Te Morsche R.H., van Heumen B.W., Nagengast F.M., Peters W.H. (2014). Over-expression of COX-2 mRNA in colorectal cancer. BMC Gastroenterol..

[24] Yin T.F., Wang M., Qing Y., Lin Y.M., Wu D. (2016). Research progress on chemopreventive effects of phytochemicals on colorectal cancer and their mechanisms. World J. Gastroenterol..

[25] Goel A., Boland C.R., Chauhan D.P. (2001). Specific inhibition of cyclooxygenase-2 (COX-2) expression by dietary curcumin in HT-29 human colon cancer cells. Cancer Lett..

[26] Lee Y.-K., Park S.Y., Kim Y.-M., Park O.J. (2009). Regulatory E-ect of the AMPK-COX-2 Signaling Pathway in Curcumin-Induced Apoptosis in HT-29 Colon Cancer Cells. Ann. N. Y. Acad. Sci..

[27] Naugler W.E., Karin M. (2008). NF-kB and cancer-identifying targets and mechanisms. Curr. Opin. Genet. Dev..

[28] Sandur S.K., Deorukhkar A., Pandey M.K., Pabón A.M., Shentu S., Guha S., Aggarwal B.B., Krishnan S. (2009). Curcumin modulates the radiosensitivity of colorectal cancer cells by suppressing constitutive and inducible NF-kB activity. Int. J. Radiat. Oncol. Biol. Phys..

[29] Collett G.P., Campbell F.C. (2006). Overexpression of p65/RelA potentiates curcumin-induced apoptosis in HCT-116 human colon cancer cells. Carcinogenesis.

[30] Shang S., Hua F., Hu Z.W. (2017). The regulation of beta-catenin activity and function in cancer: Therapeutic opportunities. Oncotarget.

[31] Valenta T., Hausmann G., Basler K. (2012). The many faces and functions of β-catenin. EMBO J..

[32] Narayan S. (2004). Curcumin, amulti-functional chemopreventive agent, blocks growth of colon cancer cells by targeting β-catenin-mediated transactivation and cell-cell adhesion pathways. J. Mol. Histol..

[33] Park C.H., Hahm E.R., Park S., Kim H.K., Yang C.H. (2005). The inhibitory mechanism of curcumin and its derivative against β-catenin/Tcf signaling. FEBS Lett..

[34] Watson A.J.M. (2004). Apoptosis and colorectal cancer. Gut.

[35] Song G., Mao Y.B., Cai Q.F., Yao L.M., Ouyang G.L., Bao S.D. (2005). Curcumin induces human HT-29 colon adenocarcinoma cell apoptosis by activating p53 and regulating apoptosis-related protein expression. Braz. J. Med. Biol. Res..

[36] Moragoda L., Jaszewski R., Majumdar A.P.N. (2001). Curcumin induced modulation of cell cycle and apoptosis in gastric and colon cancer cells. Anticancer Res..

[37] Su C.C., Lin J.G., Li T.M., Chung J.G., Yang J.S., Ip S.W., Lin W.C., Chen G.W. (2006). Curcumin-induced apoptosis of human colon cancer COLO-205 cells through the production of ROS, Ca2+ and the activation of caspase 3. Anticancer Res..

[38] Danial N.N., Korsmeyer S.J. (2004). Cell death: Critical control points. Cell.

[39] Jung E.M., Lim J.H., Lee T.J., Park J.W., Choi K.S., Kwon T.K. (2005). Curcumin sensitizes tumor necrosis factor-related apoptosis-inducing ligand (TRAIL)-induced apoptosis through reactive oxygen species-mediated upregulation of death receptor 5 (DR5). Carcinogenesis.

[40] Cao A.L., Li Q., Yin P.H., Dong Y., Shi H., Wang L., Ji G., Xie J., Wu D. (2013). Curcumin induces apoptosis in human gastric carcinoma AGS cells and colon carcinoma HT-29 cells through mitochondrial dysfunction and endoplasmic reticulum stress. Apoptosis.

[41] Thayyullathil F., Chathoth S., Hago A., Patel M., Galadari S. (2008). Rapid reactive oxygen species (ROS) generation induced by curcumin leads to caspase-dependent and -independent apoptosis in L929 cells. Free Radic. Biol. Med..

[42] Elizabeth K., Rao M.N.A. (1990). Oxygen radical scavenging activity of curcumin. Int. J. Pharmacol..

[43] Perkins S., Verschoyle R.D., Hill K., Parveen I., Threadgill M.D., Sharma R.A., Williams M.L., Steward W.P., Gescher A.J. (2002). Chemopreventive efficacy and pharmacokinetics of curcumin in the min/+ mouse, a model of familial adenomatous polyposis. Cancer Epidemiol. Biomarkers Prev..

[44] Park J., Conteas C.N. (2010). Anti-carcinogenic properties of curcumin on colorectal cancer. World J. Gastrointest. Oncol..

[45] McFadden R.M., Larmonier C.B., Shehab K.W., Midura-Kiela M., Ramalingam R., Harrison C.A., Besselsen D.G., Chase J.H., Caporaso J.G., Jobin C. (2015). The Role of Curcumin in Modulating Colonic Microbiota During Colitis and Colon Cancer Prevention. Inflamm. Bowel Dis..

[46] Mehta R.S., Song M., Nishihara R., Drew D.A., Wu K., Qian Z.R., Fung T.T., Hamada T., Masugi Y., da Silva A. (2017). Dietary Patterns and Risk of Colorectal Cancer: Analysis by Tumor Location and Molecular Subtypes. Gastroenterology.

[47] Guo Y., Wu R., Gaspar J.M., Sargsyan D., Su Z.Y., Zhang C., Gao L., Cheng D., Li W., Wang C. (2018). DNA methylome and transcriptome alterations and cancer prevention by curcumin in colitis-accelerated colon cancer in mice. Carcinogenesis.

[48] Peng S., Li Z., Zou L., Liu W., Liu C., McClements D.J. (2018). Enhancement of Curcumin Bioavailability by Encapsulation in Sophorolipid-Coated Nanoparticles: An in Vitro and in Vivo Study. J. Agric. Food Chem..

[49] Han W., Xie B., Li Y., Shi L., Wan J., Chen X., Wang H. (2019). Orally Deliverable Nanotherapeutics for the Synergistic Treatment of Colitis-Associated Colorectal Cancer. Theranostics.

[50] Di Leo A., Barone M., Maiorano E., Tanzi S., Piscitelli D., Marangi S., Lofano K., Ierardi E., Principi M., Francavilla A. (2008). ER-beta expression in large bowel adenomas: Implications in colon carcinogenesis. Dig. Liver Dis..

[51] Barone M., Tanzi S., Lofano K., Scavo M.P., Pricci M., Demarinis L., Papagni S., Guido R., Maiorano E., Ingravallo G. (2010). Dietary-induced ERbeta upregulation counteracts intestinal neoplasia development in intact male ApcMin/+ mice. Carcinogenesis.

[52] Liu H.P., Gao Z.H., Cui S.X., Wang Y., Li B.Y., Lou H.X., Qu X.J. (2013). Chemoprevention of intestinal adenomatous polyposis by acetyl-11-keto-beta-boswellic acid in APC(Min/+) mice. Int. J. Cancer.

[53] Cheung K.L., Khor T.O., Kong A.N. (2009). Synergistic effect of combination of phenethyl isothiocyanate and sulforaphane or curcumin and sulforaphane in the inhibition of inflammation. Pharm. Res..

[54] Girardi B., Principi M., Pricci M., Giorgio F., Iannone A., Losurdo G., Ierardi E., Di Leo A., Barone M. (2018). Chemoprevention of inflammation-related colorectal cancer by silymarin, acetyl-11-keto-β-boswellic acid, curcumin and maltodestrin enriched dietetic formulation in animal model. Carcinogenesis.

[55] Principi M., Di Leo A., Pricci M., Scavo M.P., Guido R., Tanzi S., Piscitelli D., Pisani A., Ierardi E., Comelli M.C. (2013). Phytoestrogens/insoluble fibers and colonic estrogen receptor β: Randomized, double-blind, placebo-controlled study. World J. Gastroenterol..

[56] Di Leo A., Nesi G., Principi M., Piscitelli D., Girardi B., Pricci M., Losurdo G., Iannone A., Ierardi E., Tonelli F. (2016). Epithelial turnover in duodenal familial adenomatous polyposis: A possible role for estrogen receptors?. World J. Gastroenterol..

[57] Girardi B., Pricci M., Giorgio F., Piazzolla M., Iannone A., Losurdo G., Principi M., Barone M., Ierardi E., Di Leo A. (2020). Silymarin, boswellic acid and curcumin enriched dietetic formulation reduces the growth of inherited intestinal polyps in an animal model. World J. Gastroenterol..

[58] Wang R., Wang Y., Gao Z., Qu X. (2014). The comparative study of acetyl-11-keto-beta-boswellic acid (AKBA) and aspirin in the prevention of intestinal adenomatous polyposis in APC(Min/+) mice. Drug Discov. Ther..

[59] Rajamanickam S., Velmurugan B., Kaur M., Singh R.P., Agarwal R. (2010). Chemoprevention of intestinal tumorigenesis in APCmin/+ mice by silibinin. Cancer Res..

[60] Storka A., Vcelar B., Klickovic U., Gouya G., Weisshaar S., Aschauer S., Bolger G., Helson L., Wolzt M. (2015). Safety, tolerability and pharmacokinetics of liposomal curcumin in healthy humans. Int. J. Clin. Pharmacol. Ther..

[61] Irving G.R., Howells L.M., Sale S., Kralj-Hans I., Atkin W.S., Clark S.K., Britton R.G., Jones D.J., Scott E.N., Berry D.P. (2013). Prolonged biologically active colonic tissue levels of curcumin achieved after oral administration--a clinical pilot study including assessment of patient acceptability. Cancer Prev. Res. (Phila.).

[62] Garcea G., Berry D.P., Jones D.J., Singh R., Dennison A.R., Farmer P.B., Sharma R.A., Steward W.P., Gescher A.J. (2005). Consumption of the putative chemopreventive agent curcumin by cancer patients: Assessment of curcumin levels in the colorectum and their pharmacodynamic consequences. Cancer Epidemiol. Biomarkers Prev..

[63] Carroll R.E., Benya R.V., Turgeon D.K., Vareed S., Neuman M., Rodriguez L., Kakarala M., Carpenter P.M., McLaren C., Meyskens F.L. (2011). Phase IIa clinical trial of curcumin for the prevention of colorectal neoplasia. Cancer Prev. Res. (Phila.).

[64] Cruz-Correa M., Shoskes D.A., Sanchez P., Zhao R., Hylind L.M., Wexner S.D., Giardiello F.M. (2006). Combination treatment with curcumin and quercetin of adenomas in familial adenomatous polyposis. Clin. Gastroenterol. Hepatol..

[65] Cruz-Correa M., Hylind L.M., Marrero J.H., Zahurak M.L., Murray-Stewart T., Casero R.A., Montgomery E.A., Iacobuzio-Donahue C., Brosens L.A., Offerhaus G.J. (2018). Efficacy and Safety of Curcumin in Treatment of Intestinal Adenomas in Patients with Familial Adenomatous Polyposis. Gastroenterology.

[66] Alfonso-Moreno V., López-Serrano A., Moreno-Osset E. (2017). Chemoprevention of polyp recurrence with curcumin followed by silibinin in a case of multiple colorectal adenomas. Rev. Esp. Enferm. Dig..

[67] Howells L.M., Iwuji C.O.O., Irving G.R.B., Barber S., Walter H., Sidat Z., Griffin-Teall N., Singh R., Foreman N., Patel S.R. (2019). Curcumin Combined with FOLFOX Chemotherapy Is Safe and Tolerable in Patients with Metastatic Colorectal Cancer in a Randomized Phase IIa Trial. J. Nutr..

[68] Irving G.R., Iwuji C.O., Morgan B., Berry D.P., Steward W.P., Thomas A., Brown K., Howells L.M. (2015). Combining curcumin (C3-complex, Sabinsa) with standard care FOLFOX chemotherapy in patients with inoperable colorectal cancer (CUFOX): Study protocol for a randomised control trial. Trials.

[69] Greil R., Greil-Ressler S., Weiss L., Schönlieb C., Magnes T., Radl B., Bolger G.L., Vcelar B., Sordillo P.P. (2018). A phase 1 dose-escalation study on the safety, tolerability and activity of liposomal curcumin (Lipocurc™) in patients with locally advanced or metastatic cancer. Cancer Chemother. Pharmacol..

[70] Soleimani V., Sahebkar A., Hosseinzadeh H. (2018). Turmeric (Curcuma longa) and its major constituent (curcumin) as nontoxic and safe substances: Review. Phytother. Res..

[71] Ganiger S., Malleshappa H.N., Krishnappa H., Rajashekhar G., Rao V.R., Sullivan F. (2007). A two generation reproductive toxicity study with curcumin, turmeric yellow, in wistar rats. Food Chem. Toxicol..

[72] Rahmani S., Asgary S., Askari G., Keshvari M., Hatamipour M., Feizi A., Sahebkar A. (2016). Treatment of non-alcoholic fatty liver disease with Curcumin: A randomized placebo-controlled trial. Phytother Res..

[73] Amin F., Islam N., Anila N., Gilani A.H. (2015). Clinical efficacy of the co-administration of turmeric and black seeds (Kalongi) in metabolic syndrome—A double blind randomized controlled trial—TAK-MetS trial. Complement. Ther. Med..

[74] Chuengsamarn S., Rattanamongkolgul S., Phonrat B., Tungtrongchitr R., Jirawatnotai S. (2014). Reduction of atherogenic risk in patients with type 2 diabetes by curcuminoid extract: A randomized controlled trial. J. Nutr. Biochem..

[75] Maithili Karpaga Selvi N., Sridhar M.G., Swaminathan R.P., Sripradha R. (2015). Efficacy of turmeric as adjuvant therapy in type 2 diabetic patients. Indian. J. Clin. Biochem..

[76] Pagano E., Romano B., Izzo A.A., Borrelli F. (2018). The clinical efficacy of curcumin-containing nutraceuticals: An overview of systematic reviews. Pharmacol. Res..

[77] Medina-Caliz I., Garcia-Cortes M., Gonzalez-Jimenez A., Cabello M.R., Robles-Diaz M., Sananbria-Cabrera J., Sanjuan-Jimenez R., Ortega-Alonso A., Garcia-Munoz B., Mereno I. (2018). Herbal and Dietary Supplement-Induced Liver Injuries in the Spanish DILI Registry. Clin. Gastroenterol. Hepatol..

[78] Imam Z., Khasawneh M., Jomaa D., Iftikhar H., Sayedahmad Z. (2019). Drug Induced Liver Injury Attributed to a Curcumin Supplement. Case Rep. Gastrointest. Med..

